# Medical Students’ Perspective and Knowledge of Asymptomatic Hyperuricemia and Gout Management: A Cross-Sectional Study

**DOI:** 10.3390/healthcare9121639

**Published:** 2021-11-26

**Authors:** Sanja Zuzic Furlan, Doris Rusic, Marko Kumric, Josko Bozic, Marino Vilovic, Tina Vilovic, Marko Rada, Venija Cerovecki, Marion Tomicic

**Affiliations:** 1 Department of Family Medicine, University of Split School of Medicine, 21000 Split, Croatia; sanja.zuzic@dz-sdz.hr (S.Z.F.); tvilovic@mefst.hr (T.V.); ravnatelj@dz-sdz.hr (M.R.); 2 Department of Family Medicine, Split-Dalmatia County Health Center, 21000 Split, Croatia; 3 Department of Pharmacy, University of Split School of Medicine, 21000 Split, Croatia; doris.rusic@mefst.hr; 4 Department of Pathophysiology, University of Split School of Medicine, 21000 Split, Croatia; marko.kumric@mefst.hr (M.K.); josko.bozic@mefst.hr (J.B.); marino.vilovic@mefst.hr (M.V.); 5 Department of Family Medicine, University of Zagreb School of Medicine, 10000 Zagreb, Croatia; venija.cerovecki@mef.hr

**Keywords:** asymptomatic hyperuricemia, gout, medical students, questionnaire, knowledge

## Abstract

The prevalence and incidence of gout doubled from 1990 to 2017. Therefore, we can expect that a number of doctors have come across a patient with gout in their daily practice. Hence, we wanted to investigate how familiar our medical students, as future medical professionals, are with gout. This cross-sectional survey included Medical Studies students from the two largest universities in Croatia: the University of Split School of Medicine, and the University of Zagreb School of Medicine, and included a total of 221 fifth or sixth year medical students. Most students gave correct answers to questions about treatment approach and non-pharmacological interventions in asymptomatic hyperuricemia (>80%). Less than 3% of all students agreed they knew enough about care for patients with asymptomatic hyperuricemia, whereas almost 15% thought they were well familiar with care for gout patients. Less than 8% of students considered their school education adequate on both topics, and less than 2% were aware of the existence of EULAR guidelines. Physicians lacking in the latest knowledge on the pathophysiology of gout, the influence of lifestyle, and genetic factors limits their ability to properly manage gout. With increasing prevalence, gout should be more represented in medical students’ education.

## 1. Introduction

Gout is considered to be the most common inflammatory arthritis worldwide [1]. It is a condition that develops when monosodium urate crystals deposit in joints. This is often proceeded by a chronic elevation of uric acid levels. Gout and hyperuricemia are closely related, yet distinct entities. In fact, as many as 90% of patients with hyperuricemia do not have clinical features of gout [2].

Although uric acid is an endogenous antioxidant, studies link high serum uric acid levels with a number of conditions, including hypertension and cardiovascular disease, as well as metabolic syndrome and diabetes [3,4,5,6]. A large Korean study on more than 11,000 participants found a dose–response relationship between uric acid levels and decreased kidney function in both men and women [7]. The causal relationship and mechanisms connecting gout, hyperuricemia, and various comorbidities are complex, and not fully understood, with contradicting reports and studies [8,9,10]. However, different comorbidities are more commonly found in patients with gout compared to the population without gout. These include cardiovascular diseases (i.e., myocardial infarction, stroke, and heart failure), renal disease (i.e., nephrolithiasis and chronic kidney disease), and metabolic syndrome (obesity, diabetes, and hypertension) [8,11,12]. Moreover, uric acid cut-off values for cardiovascular mortality are lower than general ones [13]. Another challenge in estimating gout and hyperuricemia risks is the fact that women and ethnical minorities are underrepresented in controlled clinical trials testing serum uric acid lowering drugs [14]. Urbanization is shown to be another factor linked with hyperuricemia and related risks [15]. Moreover, worse health-related quality of life referring to gout medication adverse effects was found among younger gout patients living in urban areas [16].

Hyperuricemia is a central feature in the pathogenesis of gout. Serum urate concentration is a complex phenotype influenced by both genetic and environmental factors, as well as the interactions between them. Hyperuricemia results from an imbalance between endogenous production and excretion of urate. However, the main cause of hyperuricemia is reduced renal excretion of urate [17]. Accumulating evidence suggests that the net amount of excreted uric acid is regulated mainly by urate transporters. Decreased extra-renal urate excretion caused by ABCG2 dysfunction is a common mechanism of hyperuricemia. ABCG2 genetic variants, common and rare, have been shown to have stronger effects on the risk of hyperuricemia than major environmental risk factors, such as obesity and heavy drinking. The most common dysfunction variant, rs2231142 (p.Q141K), increases the risk of gout and hyperuricemia, and significantly influences the age of onset of gout [18,19,20]. Non-synonymous allelic variants, common and rare, of ABCG2, had a significant effect on the earlier onset of gout and the presence of a familial gout history, and ABCG2 dysfunction was reported as a strong independent risk for paediatric-onset hyperuricemia/gout [21,22]. Moreover, a significant association between rs2231142 and an increased risk of a poor response to allopurinol has been described. Taken together, ABCG2 is known to be a key genetic determinant regarding the onset of gout; additionally, it plays a role in the progression and severity of the disease, and is associated with a poor response to allopurinol [23,24,25].

The prevalence and incidence of gout doubled from 1990 to 2017 [1]. In 2017, estimates are that there were 41.2 million prevalent cases of gout. The burden of gout is greatest in developed countries [26]. A study investigating worldwide gout epidemiology found that the incidence of gout has risen by 37% from 1992 to 2017, the prevalence by 41%, and specific disability-adjusted life years (DALYs) by 99%. All three were found to be substantially higher in men compared to women [27]. According to a study in Lancet, the prevalence of gout has risen 26.4% from 2005 to 2015 [28]. The distribution of gout is uneven, and developed countries tend to carry more burden [29]. The prevalence of gout in United States is estimated at 3.2% (5.2% in men, and 2.7% in women) [30]. The prevalence of hyperuricemia in mainland China is estimated to be around 13%, and of gout around 1% [31]. Moreover, the prevalence of hyperuricemia among adolescents in China is surprisingly high [32].

In terms of public health, gout is perceived as less serious and important than ischemic heart disease, diabetes, or chronic kidney disease. Although being a lower priority illness, gout predominantly affects men in their productive years, further deepening the negative effects of gout on society in general [12,33]. According to an Australian study, the average age at diagnosis is 42 years. One third of study participants reported economic hardship. As many as 76% of patients reported that gout had affected them at work [33]. The burden of gout is maybe best described in a study by Chua et al. in which more than 50% of studied patients perceived their disease as severe or very severe [12]. As many as 70% of patients may suffer uncontrolled gout, adversely affecting their performance in terms of self-care, mobility, and usual activities. Both patients with adequately controlled and uncontrolled gout miss work time, 3.6 vs. 4.5%, respectively. Moreover, patients with uncontrolled gout have greater impairment in work productivity, and more activity impairment compared to patients with well-controlled gout [34]. The burden of gout on society is stressed with the fact that it mostly affects working-age individuals [33]. It is obvious that gout may add considerably to health care costs, as it is related to frequent hospitalizations and emergency room visits, loss of productivity, and disability [1].

Most patients with well-controlled gout are managed in primary care [12,35]. They may be attended by medical graduates, internists, and orthopedic surgeons [36]. Moreover, in a study investigating the management of gout by rheumatologists, in 58.7% of cases, gout patients were referred from a general practitioner, and around 10% of them had seen two other doctors prior to the rheumatologist consult [37]. Taking this into consideration, the education of primary care physicians and medical students is of paramount importance [1]. We can expect that a number of doctors, regardless of their specialty, have come across a patient with gout in their daily practice. Moreover, studies show that 40% of patients consider that there is a need for greater awareness about the impact and severity of gout [38]. Hence, we wanted to investigate how familiar our medical students, as future medical professionals, are with gout.

## 2. Materials and Methods

### 2.1. Participants

This cross-sectional survey included Medical Studies students from the two largest Universities in Croatia: the University of Split School of Medicine (University A), and the University of Zagreb School of Medicine (University B). In order to assess knowledge regarding hyperuricemia and gout, students from last two years of studies (fifth and sixth) were eligible for participation, due to them having already passed major clinical courses. The survey was distributed online via a Google Docs^®^ link containing a comprehensive questionnaire during June and July of 2021. A link with an appropriate explanation of the study was shared in social media groups and mailing lists containing exclusively students from the mentioned Medical Studies years, and all potential questions could have been asked online. The survey was completely voluntary, anonymous, and without any kind of compensation, and the included questions did not collect personal information that could reveal students’ identities. The study was done under all assumptions from the Helsinki Declaration, and was approved by the Ethics Committee of the University of Split School of Medicine. Submitted responses by participants were considered as collected informed consent.

### 2.2. Survey

The questionnaire used was developed by three family physician specialists at the Department of Family medicine, the University of Split School of Medicine, after rigorous research of the available literature regarding hyperuricemia and gout. It consisted of three separate parts, where the first part included four items collecting students’ general information (sex, attended university and year of study, sources of information on gout).

The second part of the survey investigated students’ knowledge regarding various characteristics and inter-relations of hyperuricemia and gout. The questionnaire was already used in the population of family physicians in the Republic of Croatia [39], and it consisted of 16 multiple choice questions (MCQs), with only one correct answer among five offered in each of the questions. The draft version of the questionnaire consisted of 24 items; however, after additional review by two family medicine specialists, eight questions were disregarded due to the low intelligibility and potentially ambiguous answers.

The third part of the survey investigated students’ attitudes regarding the management of hyperuricemia and gout, and the final version consisted of nine different items in which agreement could be measured through a 5-point Likert scale (from fully agree to fully disagree). Statements were based on a previous publication that investigated attitudes on hyperuricemia and gout in a population of family physicians [39]; however, they were carefully adapted and changed to be more suitable for the student population. The draft version consisted of 14 items, from which five statements were disregarded after additional review by two family medicine specialists, due to low readability and unsuitability for the student population.

### 2.3. Statistical Analysis

Statistical analysis was performed using MedCalc statistical software (version 19.1.2; MedCalc Software, Ostend, Belgium). Categorical variables were presented as whole numbers and percentages, with a chi-squared test and Fisher’s exact test used for measuring statistical differences. Furthermore, total knowledge score was presented as median and interquartile range, with a Mann–Whitney U test used to measure statistical differences between different universities, study years, and sources of education. Statistical significance was set at a two-tailed *p* value less than 0.05.

## 3. Results

This study included a total of 221 medical students from the two largest universities in Croatia. There were 139 (62.9%) women and 82 (37.1%) men. The study included fifth (47.1%) and sixth year (52.9%) medical students. Most students used either school (37.6%), or school and the internet (38.9%) as sources of education on gout. The internet and media were more preferred at university A (13.0%) compared to university B, and school, internet, and medical journals were more often sources of information for students at university B compared to students from university A (Table 1).

**Figure 1:**
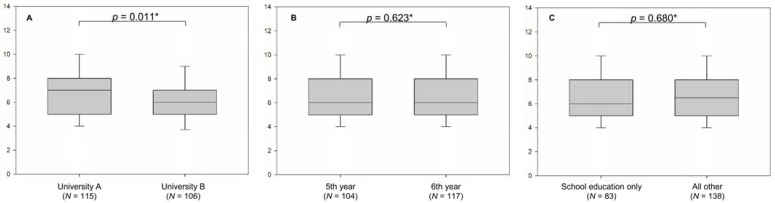
Students’ knowledge score about gout and asymptomatic hyperuricemia relative to university (**A**), study year (**B**), and source of education (**C**). * Mann–Whitney U test.

Median value for knowledge was significantly greater among students from university A (7.00 IQR 5.25–8.00) compared to students at university B (6.00, IQR 5.00–7.00, *p* = 0.011), as presented in Figure 1A. There was no significant difference in knowledge when students were compared relative to their study year (*p* = 0.0623). Both sixth and fifth year students scored a median of 6.00 (IQR 5.00–8.00), Figure 1B. Students that stated that school was their sole source of education of gout had a median of 6.00 (IQR 5.00–8.00), whereas all other students had a median of 6.50 (IQR 5.00–8.00). This difference was not significant (*p* = 0.680) (Figure 1C).

Most students gave correct answers to questions about treatment approach and non-pharmacological interventions in asymptomatic hyperuricemia (>80%). Around 50% of students correctly identified treatment for gout flares. Students mostly struggled with the definition of asymptomatic hyperuricemia and treatment goals (Table 2).

A significant difference between universities was observed for questions about treatment approach in asymptomatic hyperuricemia (*p* = 0.024), defining asymptomatic hyperuricemia as a risk factor (*p* = 0.042), treatment of gout flares (*p* = 0.004), and second line treatment for asymptomatic hyperuricemia (*p* = 0.004), with students from university A giving the correct answer significantly more often compared to students from university B (Table 2).

When observed relative to study year, significantly more students of the sixth year knew the treatment approach in asymptomatic hyperuricemia (89.7 vs. 76.0%, *p* = 0.006). However, significantly more fifth year students knew drug classes for the treatment of hyperuricemia in Croatia (84.6 vs. 67.5%, *p* = 0.003), and to identify asymptomatic hyperuricemia as a risk factor (44.2 vs. 21.4%, *p* ˂ 0.001; Table 3).

Less than 3% of all students agreed they knew enough about care for patients with asymptomatic hyperuricemia, whereas almost 15% thought they were well familiar with care for gout patients. Less than 8% of students considered their school education adequate on both topics, and less than 2% were aware of the existence of the European Alliance of Associations for Rheumatology (EULAR) guidelines. Students did not consider physicians to be quite successful in affecting their patients’ lifestyle habits. The use of guidelines in clinical practice, as well as national referent values for serum uric acid, are considered important for gout management among most students. Personal clinical experience for the management of these patients was considered important by less than 6% of students (Table 4).

## 4. Discussion

Misperception of gout may lead to late diagnosis and more comorbidities associated with it in the population. It is interesting to observe that although most students correctly identified treatment approaches in asymptomatic hyperuricemia (83.3%), only around 2% agreed that they knew enough about care for patients with asymptomatic hyperuricemia. Moreover, in a different study, undergraduates (28.6%) preferred referring gout patients to rheumatologists, but most postgraduates (71.4%) preferred managing the patients with gout themselves [36]. Students did not differ significantly among study years or sources of education in knowledge scores. However, in research among primary care physicians in Croatia that used the same questionnaire, physicians that read at least one scientific paper on the topic of gout scored significantly higher than those who did not [39].

Less than 2% of students stated they were familiar with the EULAR guidelines. Interestingly, almost 90% of students stated guidelines should be used in everyday practice. This proves that gout is perceived as “less important” in medical schools, and guidelines for such conditions are not mentioned through education. Less than 10% of students reported satisfaction with education about the topic so far. This is in line with poor education satisfaction among new medical graduates on gout [40]. Moreover, students did not consider personal experience as valuable in the management of gout patients. This was confirmed in a previous study in which the work experience of primary care physicians did not follow their knowledge on gout [39].

More than 75% of students knew the drugs for treatment of hyperuricemia registered in Croatia, and around 50% of students correctly identified treatment for gout flares; however, only 25.8% knew second line treatment for hyperuricemia. In a previous study among primary care physicians in Croatia, drugs were correctly identified by as many as 75% of them in all three cases. The questions about pathophysiology of hyperuricemia were better answered by students than physicians [39].

In a US study, 14.4% internists and 9.6% family practitioners reported awareness about gout treatment recommendations, and less than 50% of all participants chose the optimal medication treatment [41]. In a study among Moroccan rheumatologists, 40% routinely applied the EULAR gout classification criteria, whereas 12.4% did not know them [26]. In Nepal, 65.8% were aware of the latest guidelines [36]. In Saudi Arabia, less than a third of primary care physicians showed adequate knowledge on asymptomatic hyperuricemia [42].

The cut-off value of serum uric acid was correctly identified by a third of students. For comparison, in a Russian study on primary care physicians, less than half of respondents knew the exact cut-off value of urates, and the gold standard for the diagnosis of gout. Results were better among rheumatologists, with 90% of them identifying the gold standard for gout diagnosis, and 50% knowing the latest cut-off values of urates [43].

In an Austrian study on primary care physicians, more than 90% recognised increased cardiovascular risk in gout, and more than 90% considered diet and lifestyle as important factors in gout management. However, less than a quarter gave accurate answers on treatment recommendation, and around 60% were somewhat in line with the guidelines [35]. Around 36% of students that participated in this study knew the expected effects of lifestyle interventions on lowering hyperuricemia. Weight gain was confirmed to be a significant risk factor for gout on over 11,000 individuals. Lifestyle interventions, such as weight maintenance, may lower the risk for developing gout [44]. As little as 6% thought that physicians are successful in changing their patients’ habits. Poor physician- patient relationships in gout management are well documented. Physicians tend to believe gout has a moderate impact on emotions and life, and only a few routinely offer lifestyle advice to patients with gout [45]. In Australia, the prevalence of gout varies from 0.8% (self-reported) to 6.8% (self-reported doctor diagnosed gout), showing a poor understanding of the condition among patients [46,47]. Moreover, there are studies reporting discordance between patients and physicians on a presence of a gout flare, with a rate as high as 30% [48]. Furthermore, patients report poor follow-up and disease monitoring from their physicians, as well as a lack of discussion about treatment options [38]. Patients tend to take prescribed treatments transiently [12]. Low compliance to treatment is among the greatest challenges to reaching treatment goals. Results from a large study in Italy indicate a lack of awareness about the increasing prevalence of gout among health-care professionals. Moreover, GPs and specialists were lacking in the latest knowledge on the pathophysiology of gout, the influence of lifestyle, and genetic factors, limiting their ability to properly manage gout [49].

Research showed that 71% of patients considered their gout to be uncontrolled, and 60% reported a lack of knowledge on the condition. More than half of studied patients reported that gout affected their ability to walk, 43% reported changes in mental health and mood, and a quarter reported difficulties in the relationship with their partner. The adverse effects of gout on sexual and personal relationships were more prominent among younger patients. Furthermore, younger patients have a greater time to diagnosis, and this may negatively affect their future health. Moreover, 25% reported that a family member has retired or got fired due to gout [38]. Illness should be observed not only by its seriousness and mortality risk, but also as its effect on quality of life, cost for health care systems and individuals, and associated comorbidities and their effects.

The prevalence of comorbidities increases with the duration of gout diagnosis. Large cohorts on gout patients showed that cardiovascular disease accounts for more than half of the deaths, and that cardiovascular mortality follows gout severity. A number of drugs used to treat comorbidities in gout have various effects on hyperuricemia and gout flares [6,50]. As well, a number of common comorbidities may limit treatment options for gout management and gout flares. Furthermore, gout management should include screening for renovascular diseases and risk factors [50]. Innovative learning strategies, such as live patient encounters, may facilitate students’ learning process of complex diseases [51].

Better utilisation of primary care physicians, maybe with additional staff, such as nursing staff, for patient education, may shorten time to diagnosis, and improve patient care and disease management, and maintain treatment goals. All off these efforts would likely result in reduced hospital admissions and improved quality of life in these patients, meaning more working hours and less risk for comorbidities, resulting in substantial savings for society. Health care professionals’ dedication to a disease and its management is influenced by their perception of the burden of disease [45]. A Russian study demonstrated low awareness of gout in primary care doctors. Poor ability to recognise gout in primary care may additionally prolong time to diagnosis [43]. Preparing future generations may condense time to diagnosis, especially for “unexpected” young patients [38].

This study is not without limitations. Although an interesting finding, we do not consider the differences in students’ knowledge relative to their university an indicator of a discrepancy in teaching standards between universities. Although medicine is a regulated profession in the EU, slight differences may exist between the two universities in teaching. Furthermore, the multiple-choice questions in the questionnaire may have contributed to somewhat biased answers. For example, in the question “Choose a drug that raises plasma urate levels”, possible answers were ampicillin, fenofibrate, atorvastatin, losartan, and furosemide. However, diuretics are in fact the most frequently prescribed drugs that could determine uric acid increase [52]. This study was conducted only among students in Croatia, and this may limit the generalizability of the findings. However, we have found a single study on new medical graduates, and, therefore, consider our study to be of great value for medical education and gout management.

## 5. Conclusions

Misperception of gout may lead to late diagnosis and more comorbidities associated with it in the population. This study showed that students are not familiar with gout management guidelines, and very few considered their school education about hyperuricemia and gout adequate. Medical students’ education should cover diseases that are increasing in prevalence, underdiagnosed, poorly recognised, and present a substantial burden to healthcare systems, such as gout. Preparing future generations may condense time to diagnosis for these patients and improve outcomes, resulting in less expense for healthcare systems.

## Figures and Tables

**Table 1:** Characteristics of study participants.

Variable	University A (*N* = 115)	University B (*N* = 106)	*p* Value *	All (*N* = 221)
Sex				
Men	38 (33.0)	44 (41.5)	0.194	82 (37.1)
Women	77 (67.0)	62 (58.5)		139 (62.9)
Year of study 5th	57 (49.6)	47 (44.3)	0.438	104 (47.1)
Year of study 6th	58 (50.4)	59 (55.7)		117 (52.9)
Sources of education				
School	39 (33.9)	44 (41.5)	0.024	83 (37.6)
Internet and media	15 (13.0)	3 (2.8)		18 (8.1)
Medical journals	0 (0.0)	0 (0.0)		0 (0.0)
School and internet	47 (40.9)	39 (36.8)		86 (38.9)
School and medical journals	1 (0.9)	3 (2.8)		4 (1.8)
Internet and medical journals	2 (1.7)	0 (0.0)		2 (0.9)
School, internet, medical journals	11 (9.6)	17 (16.0)		28 (12.7)

* Chi-squared test or Fisher’s exact test; Data are presented as whole number (percentage).

**Table 2:** Correct answers to knowledge questions relative to students’ university.

Question	University A (*N* = 115)	University B (*N* = 106)	*p* Value *	All (*N* = 221)
Q1. Treatment approach in asymptomatic hyperuricemia	102 (88.7)	82 (77.4)	0.024	184 (83.3)
Q2. Non-pharmacological interventions for hyperuricemia	95 (82.6)	83 (78.3)	0.420	178 (80.5)
Q3. Drug classes for treatment of hyperuricemia registered in Croatia	89 (77.4)	78 (73.6)	0.512	167 (75.6)
Q4. Relationship of asymptomatic hyperuricemia and gout	72 (62.2)	64 (60.4)	0.734	136 (61.5)
Q5. Treatment of gout flares	68 (59.1)	42 (39.6)	0.004	110 (49.8)
Q6. Diagnostic procedure for confirmation of gout diagnosis	46 (40.0)	53 (50.0)	0.136	99 (44.8)
Q7. The expected effect of non-pharmacological treatment options for lowering hyperuricemia	43 (37.4)	36 (34.0)	0.596	79 (35.7)
Q8. Drug for lowering hyperuricemia registered in Croatia (with reference to the most likely cause of hyperuricemia in most patients)	47 (40.9)	32 (30.2)	0.099	79 (35.7)
Q9. Identifying drugs that elevate serum uric acid levels	44 (38.3)	30 (28.3)	0.118	74 (33.5)
Q10. Asymptomatic hyperuricemia as a risk factor	44 (38.3)	27 (25.5)	0.042	71 (32.1)
Q11. Cut-off value of serum uric levels for initiation of pharmacological treatment	32 (27.8)	39 (36.8)	0.155	71 (32.1)
Q12. Most common cause of elevated urate levels	37 (32.2)	27 (25.5)	0.274	64 (29.0)
Q13. Second line of treatment of hyperuricemia	39 (33.9)	18 (17.0)	0.004	57 (25.8)
Q14. Identifying drugs that lower serum uric acid levels	27 (23.5)	26 (24.5)	0.855	53 (24.0)
Q15. The goal when treating hyperuricemia	14 (12.2)	9 (8.5)	0.371	23 (10.4)
Q16. Definition of asymptomatic hyperuricemia	6 (5.2)	9 (8.5)	0.335	15 (6.8)

* Chi-squared test or Fisher’s exact test; Data are presented as whole number (percentage).

**Table 3:** Correct answers to knowledge questions relative to students’ study year.

Question	5th Year (*N* = 104)	6th Year (*N* = 117)	*p* Value *
Q1. Treatment approach in asymptomatic hyperuricemia	79 (76.0)	105 (89.7)	0.006
Q2. Non-pharmacological interventions for hyperuricemia	79 (76.0)	99 (84.6)	0.106
Q3. Drug classes for treatment of hyperuricemia registered in Croatia	88 (84.6)	79 (67.5)	0.003
Q4. Relationship of asymptomatic hyperuricemia and gout	62 (59.6)	74 (63.2)	0.580
Q5. Treatment of gout flares	52 (50.0)	58 (49.6)	0.950
Q6. Diagnostic procedure for confirmation of gout diagnosis	44 (42.3)	55 (47.0)	0.484
Q7. The expected effect of non-pharmacological treatment options for lowering hyperuricemia	32 (30.8)	47 (40.2)	0.146
Q8. Drug for lowering hyperuricemia registered in Croatia (with reference to the most likely cause of hyperuricemia in most patients)	37 (35.6)	42 (35.9)	0.961
Q9. Identifying drugs that elevate serum uric acid levels	37 (35.6)	37 (31.6)	0.535
Q10. Asymptomatic hyperuricemia as a risk factor	46 (44.2)	25 (21.4)	<0.001
Q11. Cut-off value of serum uric levels for initiation of pharmacological treatment	30 (28.8)	41 (35.0)	0.326
Q12. Most common cause of elevated urate levels	27 (26.0)	37 (31.6)	0.355
Q13. Second line of treatment of hyperuricemia	29 (27.9)	28 (23.9)	0.504
Q14. Identifying drugs that lower serum uric acid levels	23 (22.1)	30 (25.6)	0.541
Q15. The goal when treating hyperuricemia	15 (14.4)	8 (6.8)	0.066
Q16. Definition of asymptomatic hyperuricemia	8 (7.7)	7 (6.0)	0.615

* Chi-squared test or Fisher’s exact test; Data are presented as whole number (percentage).

**Table 4:** Attitudes on management of gout and hyperuricemia.

Statement	Fully Disagree	Disagree	Unsure	Agree	Fully Agree
A1. I know enough about care for patients with asymptomatic hyperuricemia.	68 (30.8)	92 (41.6)	55 (24.9)	4 (1.8)	2 (0.9)
A2. I know enough about care for gout patients.	45 (20.4)	69 (31.2)	74 (33.5)	29 (13.1)	4 (1.8)
A3. Physicians are very successful in changing lifestyle of their patients with hyperuricemia/gout.	54 (24.4)	78 (35.3)	76 (34.4)	13 (5.9)	0 (0.0)
A4. I am familiar with the EULAR evidence-based recommendations for the management of gout.	154 (69.7)	56 (25.3)	8 (3.6)	2 (0.9)	1 (0.5)
A5. Physicians should use EULAR evidence-based recommendations for the management of gout in everyday practice.	5 (2.3)	2 (0.9)	17 (7.7)	82 (37.1)	115 (52.0)
A6. Patients with hyperuricemia/gout should mostly be approached based on personal clinical experience.	50 (22.6)	92 (41.6)	66 (29.9)	13 (5.9)	0 (0.0)
A7. I believe that guidelines for management of patients with asymptomatic hyperuricemia would be of great assistance in physicians’ everyday practice.	0 (0.0)	0 (0.0)	16 (7.2)	35 (15.8)	170 (76.9)
A8. National referent values of serum uric acid levels are important cut-off values for everyday decisions about starting pharmacotherapy in patients with asymptomatic hyperuricemia.	5 (2.3)	11 (5.0)	60 (27.1)	94 (42.5)	51 (23.1)
A9. I believe that my school education on the topic of asymptomatic hyperuricemia and gout have been adequate so far.	67 (30.3)	81 (36.7)	56 (25.3)	14 (6.3)	3 (1.4)

Data are presented as whole number (percentage).

## Data Availability

All data is available from the corresponding author upon reasonable request.
